# Experimental study on determining the degree of bone healing by wall thickness ratio analysis

**DOI:** 10.1186/s13018-024-04565-7

**Published:** 2024-01-19

**Authors:** Liangcheng Tong, Zhiwei Yang, Wei Dai, Zhongyang Sun, Junsheng Yang, Qing Xue, Ying Li

**Affiliations:** Department of Orthopedics, Air Force Hospital of Eastern Theater Command, Anhui Medical University, No. 1 Malu Road, Nanjing, 210002 Jiangsu China

**Keywords:** Bone healing judgment, Wall thickness analysis, Wall thickness ratio analysis, Quantitative judgment

## Abstract

To verify the reliability and accuracy of wall thickness ratio analysis to determine the degree of bone healing, fracture models were established with 6 beagles. X-ray, micro-CT, and CT scans were performed at 24 weeks. The healthy side and the affected side were used to simulate the three-dimensional geometric model after internal fixation, and the mesh was divided. The mean and median CT wall thickness values were obtained through the wall thickness analysis. X-ray, CT, micro-CT, and gross appearance were used to determine the degree of bone healing, which was compared with wall thickness analysis. There was a positive correlation between the average CT value and the median wall thickness. The correlation coefficient analysis of the median wall thickness ratio (R2) and healing index ratio (R3) showed a positive correlation. The results of the wall thickness ratio (R2) and the healing index ratio (R3) were used to determine bone healing, and the results were consistent with the results of the actual mechanical test and image analysis. The results of wall thickness ratio analysis were significantly correlated with the degree of bone healing. This method is simple, rapid, and practical to analyze and judge the degree of bone healing.

## Introduction

With the increase in accidental injuries, the incidence of various complex fractures is increasing. High-energy injuries, iatrogenic injuries, and other factors lead to an increasing number of nonunion and delayed union in the process of long bone healing. At present, the clinical healing of fracture tends to be subjective and general judgment and lacks objective and quantitative judgment indicators, which delays the diagnosis of poor fracture healing. How to dynamically and quantitatively analyze the degree of bone healing is an important problem in traumatic orthopedics. Fracture healing is a complex regeneration process that is similar to bone development except for the initial stage of bleeding and inflammation. With the change in the wall thickness of the bone tissue around the fracture, the bone mineral density and thickness continuously change. So the change in the bone tissue thickness in the fracture area can reflect the process of bone healing. To determine the state of bone healing, most clinicians usually rely on clinical, physical, and image examinations to determine the degree of fracture healing, but the evaluation criteria are quite different. Scholars have tried to use quantitative indices to judge bone healing considering the mechanics and imaging results, but the use of these indices has not gained popularity [1–3]. Existing studies have shown that there is a linear correlation between bone mineral density and cortical bone thickness, and the change in the bone tissue wall thickness is closely related to fracture occurrence and bone healing. The increase in the disused bone loss after fracture leads to thinning of the cortical bone, and the increase in the bone cortical thickness gradually recovers through late functional rehabilitation [4–10].

CT scanning to observe the degree of bone healing after fracture surgery is a commonly used clinical technique. The digital information from the CT scan reveals the bone segment density (CT value) [11, 12], thickness, and morphological features, reflecting that the fracture healing process continuously changes over time. Based on individual imaging data, real-time quantitative observations of the degree of bone healing have fueled research on the process of bone healing [8–10]. However, as far as the literature is aware, orthopedic surgeons currently do not have a standard method for quantitative determination of bone healing [13, 14].

To validate the reliability and accuracy of wall thickness ratio analysis for assessing the degree of bone healing, we established a model of femoral fracture in beagle dogs and used CT scan data to determine the density and stiffness of the fracture. The CT values of the callus and the wall thickness of the bone cortex were continuously determined during bone healing. The degree of bone healing was quantitatively determined by wall thickness analysis and continuous observation [4, 15–17]. After the specimens were removed in the 24th week, the experimental data were determined by compression using an electronic universal testing machine. The ratio analysis of the maximum yield force (N) between the affected side and the normal side showed that a value of more than 70% meant that the bone was healed, and a value of less than 70% meant that the bone was not healed.

## Methods

### General data and methods used with animal models

The fracture models of beagle dogs (purchased from Changzhou Beagle Experimental Animal Breeding Co., Ltd.) had the following descriptors: the animals weighed 10–15 kg, were 2–5 years old, half were male and half were female. The dog model of middle femoral fracture was established by internal fixation with a steel plate and direct fixation in the bone healing group. The model was observed continuously for 24 weeks, and the bone tissue was taken for mechanical experiments 24 weeks after the operation.

The research was reviewed and approved by Animal Ethics Review of Anhui Medical University Laboratory Animal Ethics Committee NO. LLSC 20150174 & 20150129.

### Software and hardware environment

The software used was 3 Murmatic Research 16.0 (Materialise, Belgium).

The Micro-PET/CT was equipment model Inveon Micro-PET/CT (manufacturer: SIEMENS, origin: Germany). A CMT6000 series electronic universal testing machine produced by Meters Industrial Systems (China) Co., Ltd. GE 64-row spiral CT (GE company Light Speed spiral CT).

A Dell high-performance computer was used (CPU: i7-10750H 2.60 GHz memory: 32 GB graphics card: NVIDIA GeForce RTX 2070 Super 8G operating system: Windows 10–64 bit).

### Data collection and processing

Imaging examinations (X-ray and CT scan) were performed at 1 week, 3 weeks, 6 weeks, 12 weeks, and 24 weeks. X-ray films of the hindlimbs of beagle dogs were taken. The CT scanner scanned both lower limbs. The current control system of the GE64 spiral CT automatic tube was used to scan the parameters. The distance was 0.625 mm. A total of 512 × 512 pixel CT tomographic images were obtained. The scanning voltage was 140 kV, and the exposure was 100 mAs. The general DICOM 3.0 standard format was stored.

### 3D reconstruction and wall thickness ratio analysis

The data in DICOM3.0 format of high-resolution CT are read directly by Mimics software. After localization, organization, and 3D calculation of the image, a three-dimensional geometric model (including the affected side with internal fixation, without internal fixation, and the healthy side) is generated, the triangle mesh is optimized, and the side length is optimized to 1 mm. The results of the median bone cortical wall thickness and the average CT value in the analysis results were analyzed. In a software environment, the scanned data in the same period are calculated by the ratio of the affected side to the healthy side. By analyzing the changes in the callus and bone cortex during bone healing, the median wall thickness and average CT value of the bone cortex can be calculated effectively. The wall thickness ratio (R2) is equivalent to the median wall thickness of the affected side without internal fixation divided by the median wall thickness of the healthy side. The product of the average CT gray value and the median wall thickness was defined as the healing index, and the healing index ratio (R3) is equivalent to the healing index of the affected side without internal fixation divided by the healing ratio of the healthy side.

### Analysis method of the measured load

Using the electronic universal testing machine, the two ends of the standard parts need to be flat before the compression test, and the pretension test needs to be carried out before the compression test. The tension test is carried out, the two ends of the bone are pierced, the steel wire is suspended, and the bolt is fixed on the machine head. The elasticity of the cable is reduced after pretension before testing and then testing.

### Judgment criteria

Routine judgment: The preliminary clinical results were judged by imaging clinical judgment and CT images, and the real microhealing was obtained by combining with micro-PET/CT image identification. The judging criteria of bone healing which were x-ray photos showed that the fracture line of the broken end of the fracture was blurred, the continuous callus passed through the fracture line, and there was no abnormal activity at the fracture site. Wall thickness ratio analysis which is the result of the median wall thickness ratio (R2) and healing index ratio (R3) was judged.

A patient’s bone healing and nonunion can be judged by the critical point and continuous observation on the time axis. This can effectively and intuitively develop the process of the change for bone cortical wall thickness which leads to bone healing. Thus, the healing process can be analyzed. To control the measurement error, the ratio of the median wall thickness of the simulated internal fixation side to the median wall thickness of the healthy side was selected as the evaluation index under the same scanning and threshold conditions.

The mechanical test machine load test data were taken out of the Beagle dog femoral fracture model in the 24th week [18, 19], and the results were compressed by the electronic universal testing machine. The ratio of the maximum yield force (N) of the affected side to the normal side was analyzed. More than 70% can be considered healed, and less than 70% can be considered unhealed [20].

## Results

There was a positive correlation between the average CT value and the median wall thickness. The correlation coefficient analysis of the median wall thickness ratio (R2) and healing index ratio (R3) showed a positive correlation. The results of the wall thickness ratio (R2) and the healing index ratio (R3) were used to judge bone healing. The results were consistent with the results of the actual mechanical test and an imaging evaluation (Table 1).

**Table 1:** Comparison of the results of imaging, clinical, wall thickness ratio analysis, and load test data

	Conventional judgment method		Differential ratio analysis				Residual than	
	The micro judgment	Clinical judgment	Healing Index (R3)	Judge	Median wall thickness (R2)	Judge	The compression	Judge
Dog 1	Bone healing	Bone healing	0.49	Bone healing	0.55	Bone healing	0.59	Bone healing
Dog 2	Bone healing	Bone healing	0.59	Bone healing	0.7	Bone healing	0.24	Bone healing
Dog 3	Healing	Healing	0.87	Healing	0.94	Healing	0.80	Healing
Dog 4	Bone healing	Bone healing	0.78	Bone healing	0.74	Bone healing	0.62	Bone healing
Dog 5	Healing	Healing	0.84	Healing	0.85	healing	0.71	Healing
Dog 6	Bone healing	Bone healing	0.61	Bone healing	0.61	Bone healing	0.56	Bone healing

The CT gray value data in the CT scan, the results of wall thickness ratio analysis, the conventional methods for judging bone healing, and the measured load analysis data were consistent

**Fig. 1:**
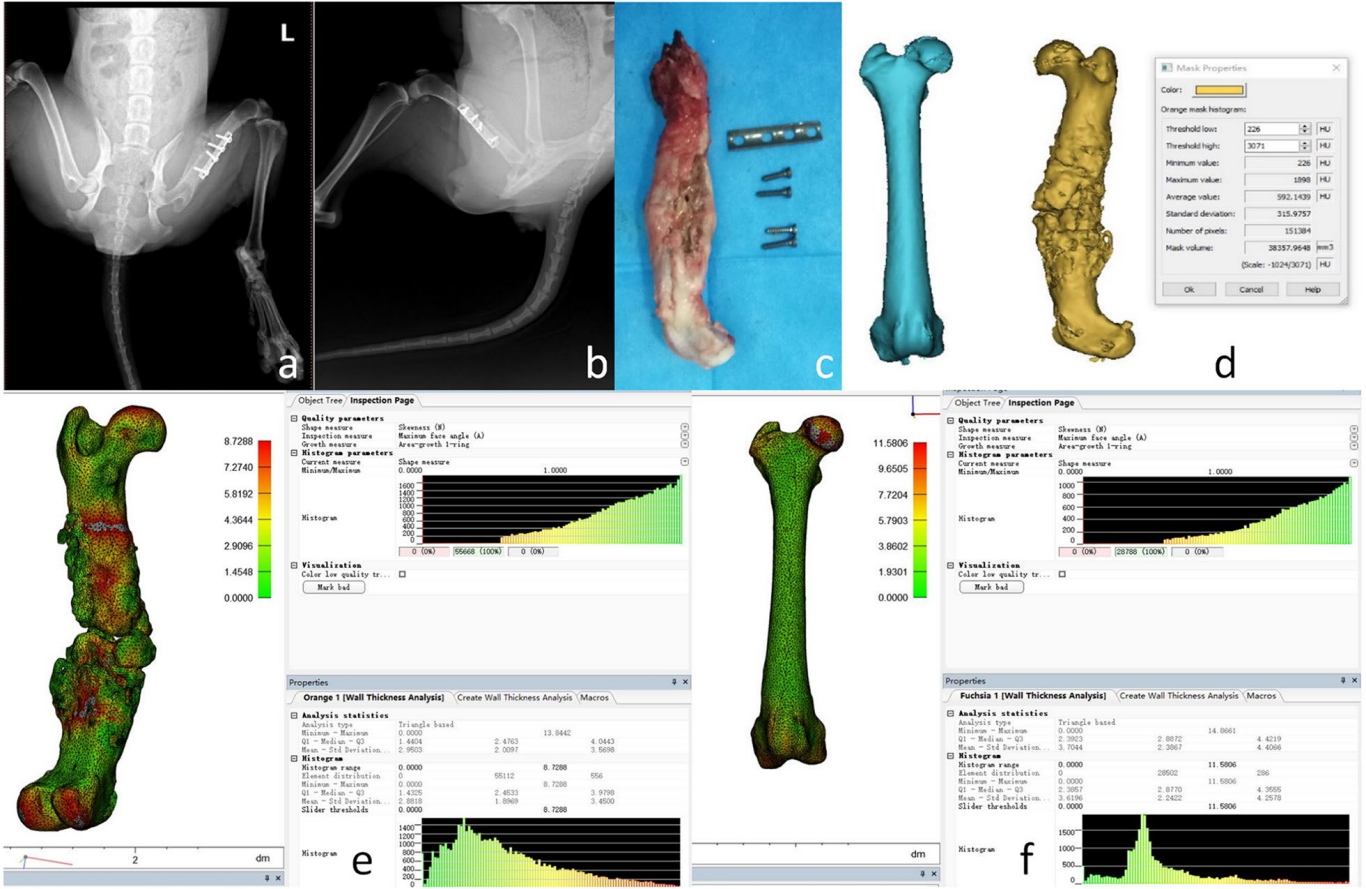
The process of wall thickness analysis of the unhealed bone group. Analysis process of the bone healing group: The model of femoral bone healing in dogs was established 24 weeks later, and the healthy side and the affected side were modeled. **a** Beagle anterograde radiographs of femur, **b** Beagle lateral radiographs of femur, **c** Physical photographs of the femoral internal fixator were taken out. **d** Three-dimensional modeling was conducted on the healthy side and the affected side using CT data to obtain CT mean value, volume, and other data. Analyze the thickness of the affected lateral wall to understand the maximum and median values. **f** Analyze the healthy lateral wall thickness to know the maximum and median. After 24 weeks, the healthy side and the affected side were modeled and analyzed by wall thickness ratio analysis, Micro-PET/CT, and mechanical test machine. It was found that the wall thickness ratio method was equal to that of the other two groups, and the femoral bone was not healed

**Fig. 2:**
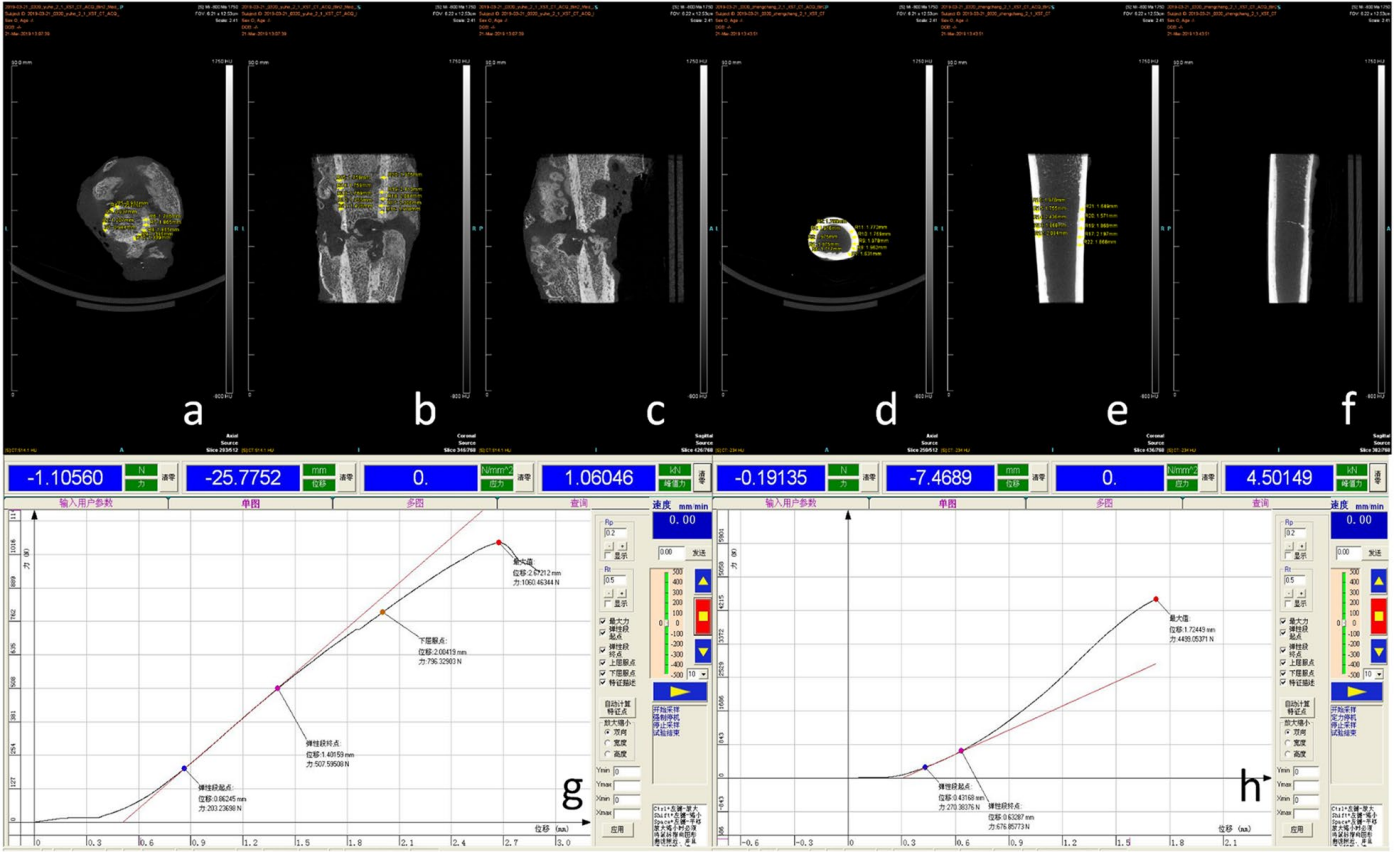
Process of mechanical analysis of unhealed bone. Analysis process of the bone healing group: The model of femoral bone healing in dogs was established 24 weeks later, and the healthy side and the affected side were modeled. **a**–**f** It is to scan the fracture through micro-CT, which can see that the fracture on the affected side is not healed, and extract data such as the cortical thickness of the healthy side. **g**, **h** Is the time-displacement curve data obtained from compressive analysis of healthy and affected bone segments on the mechanical experimental machine

**Fig. 3:**
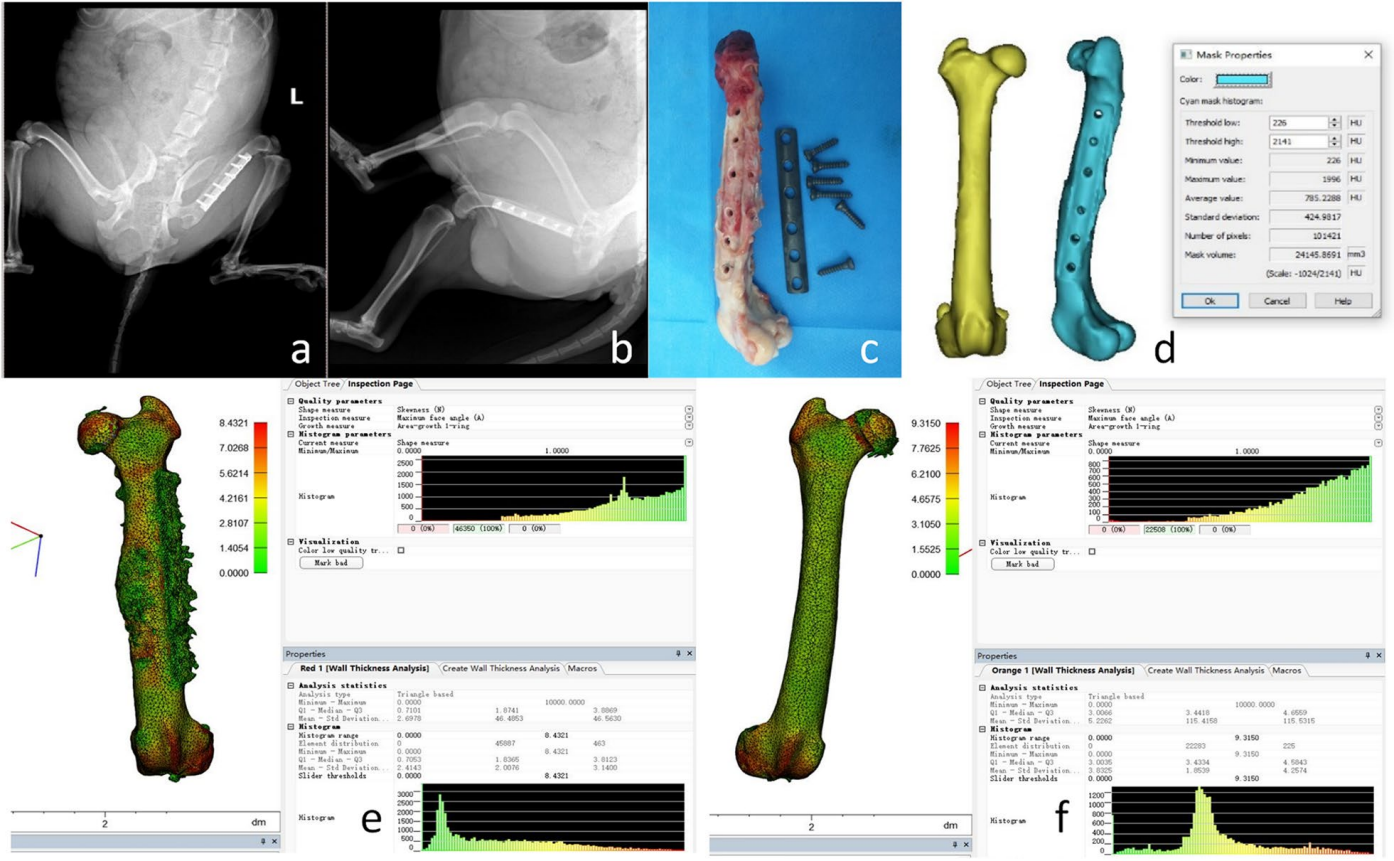
Bone healing group wall thickness analysis process. Analysis process of the bone nonunion group: The model of nonunion of the femur in dogs was established 24 weeks later, and the healthy side and the affected side were modeled. **a** Beagle anterograde radiographs of femur, **b** Beagle lateral radiographs of femur, **c** Physical photographs of the femoral internal fixator were taken out. **d** Three-dimensional modeling was conducted on the healthy side and the affected side using CT data to obtain CT mean value, volume, and other data. Analyze the thickness of the affected lateral wall to understand the maximum and median values. **f** Analyze the healthy lateral wall thickness to know the maximum and median

**Fig. 4:**
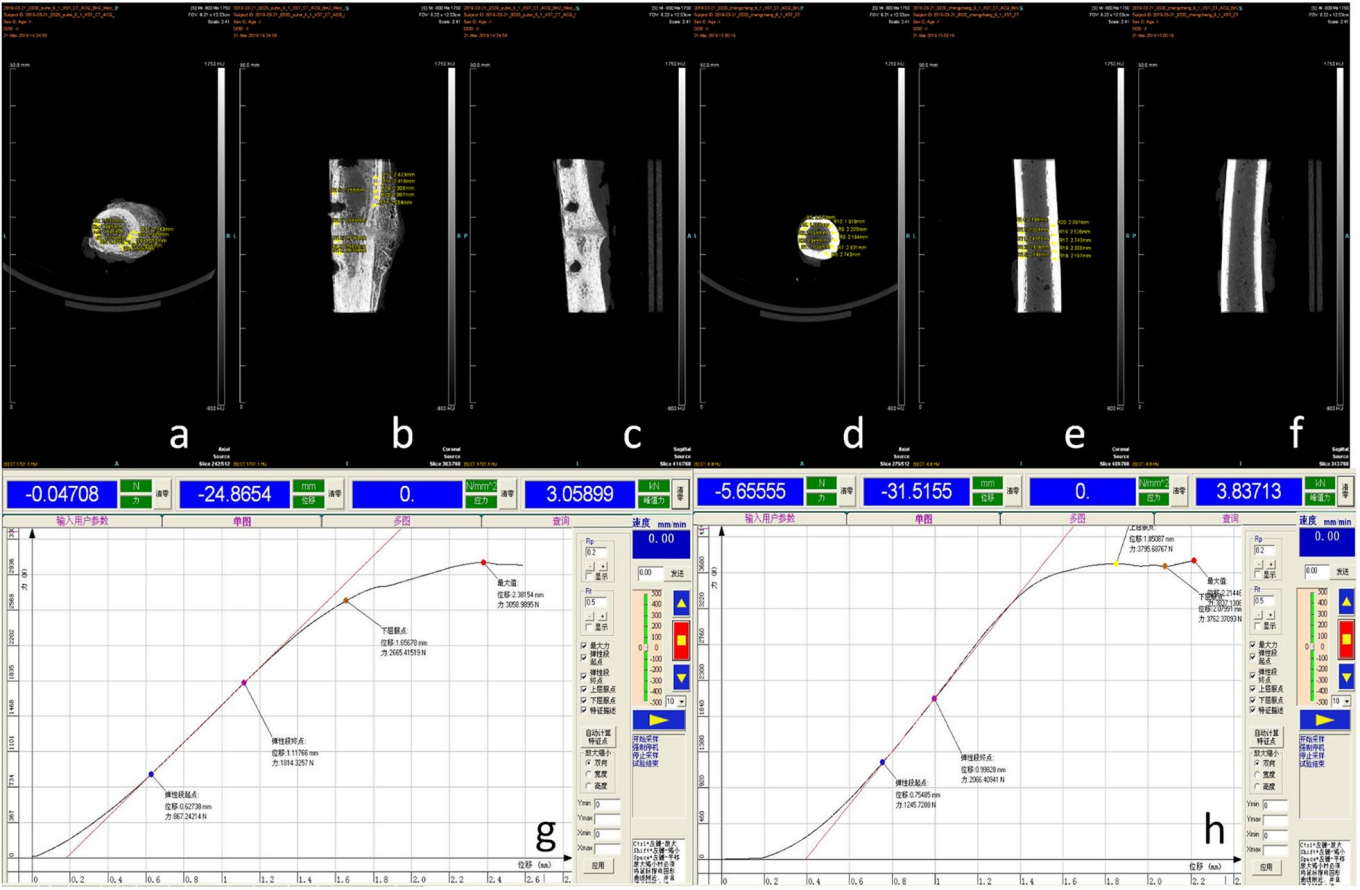
The analysis of the bone healing group. Analysis process of the bone nonunion group: The model of nonunion of the femur in dogs was established 24 weeks later, and the healthy side and the affected side were modeled. **a**–**f** Fracture healing on the affected side can be seen by micro-CT scan, and data such as cortical thickness on the healthy side can be extracted. **g**, **h** Is the time-displacement curve data obtained by compression analysis of healthy and diseased bone segments on a mechanical experimental machine

**Fig. 5:**
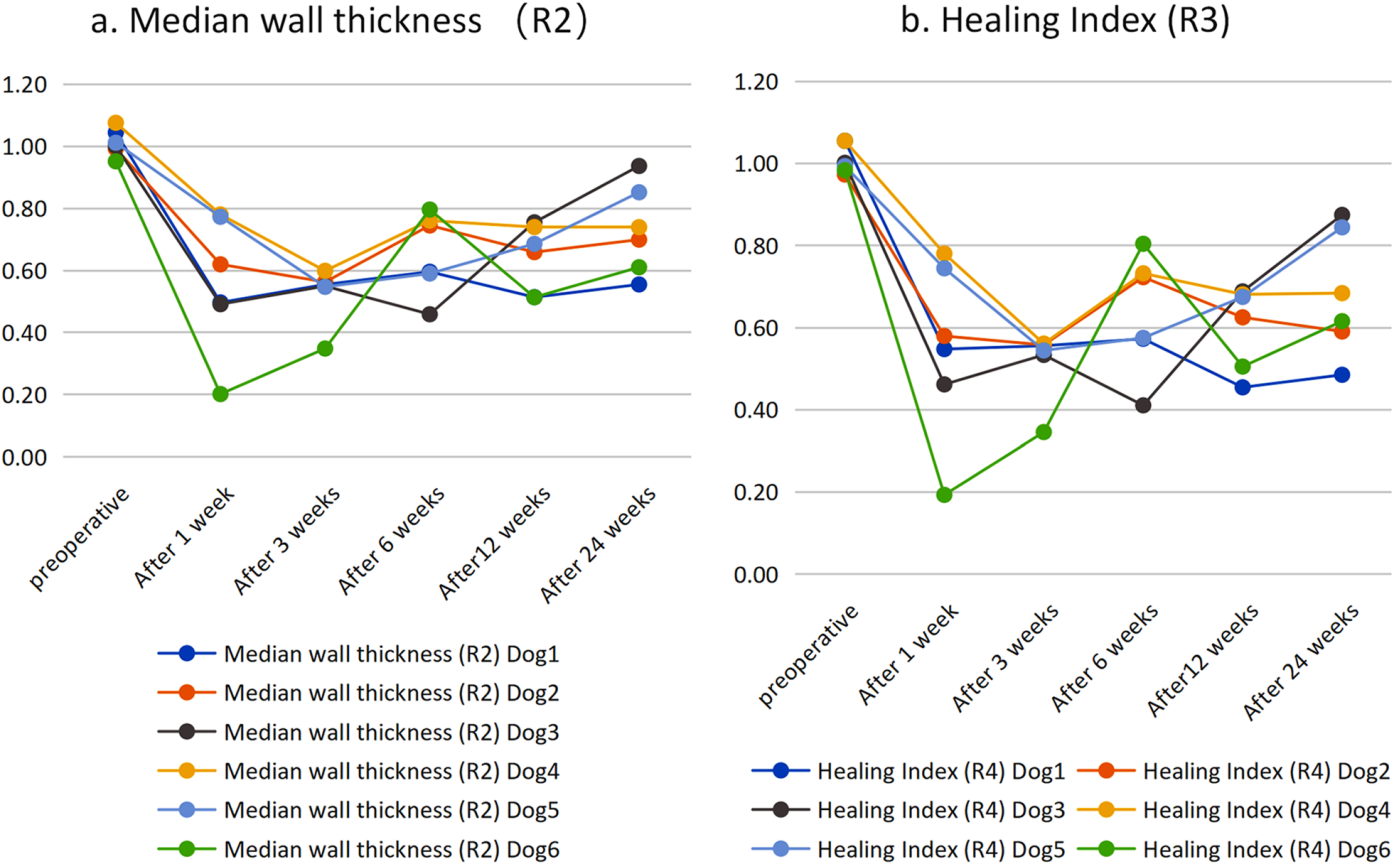
Changes in R2 and R3 of each dog at different time points

**Fig. 6:**
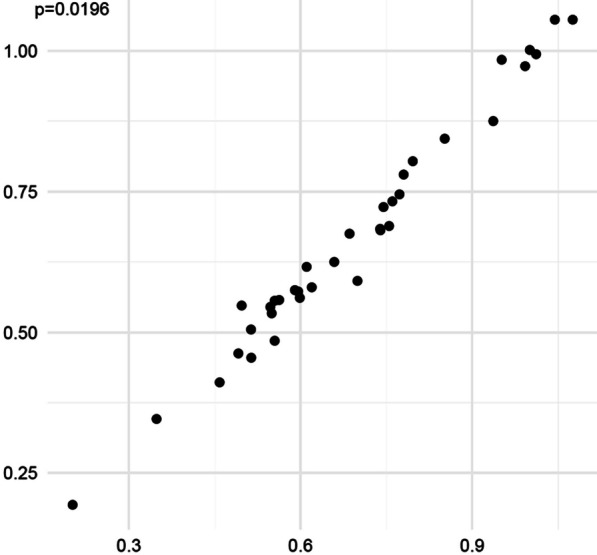
Correlation analysis. The correlation coefficient between the average CT value and the median wall thickness was *r* = 0.37, *p* = 0.0196 (*p* < 0.05), which was statistically significant and positively correlated. The condition of the canine femoral nonunion model after 24 weeks, the healthy side and the affected side were modeled respectively Analysis process of the bone healing group: X-ray, micro-CT, and CT scans were performed at 24 weeks. CT scans in DICOM3.0 standard format were stored and modeled in the software Mimics. The healthy limb and the affected side were used to simulate the 3D geometric model after internal fixation was removed, and the mesh was divided. The average CT value and median wall thickness were obtained by finite element analysis. The values measured after the simulated removal of the internal fixation on the affected side and the median wall thickness ratio (R2) and healing index ratio (R3) on the healthy side were used as indicators to observe and evaluate the degree of bone healing. After a sudden death, the bilateral hindlimbs were removed, and the degree of bone healing was judged by X-ray, CT, micro-CT, and general appearance. Standard parts of the hindlimbs were made, and the degree of bone healing was analyzed and judged. The degrees of bone healing judged by the three methods were compared and analyzed, proving that the bone is not healed

The wall thickness ratio analysis method is consistent with the actual test characteristics of the mechanical test and the imaging results. This method is also consistent with the continuous observation of the degree of bone healing on the time axis, which is consistent with the pathophysiological process of bone healing. The wall thickness ratio analysis method is simple, rapid, and practical in judging the degree of bone healing (Figs. 1, 2, 3, 4, 5 and 6).

## Discussion

Monitoring bone healing may help clinicians to better determine how well new bone is healing so that early measures can be taken to promote bone healing [21]. Therefore, how to dynamically and quantitatively analyze the degree of bone healing is an important issue in traumatic orthopedics research. In this study, the bone cortex median wall thickness and healing index ratio (R3) were continuously observed, enabling intuitive and quantitative observations of the bone healing process and evaluation of the degree of bone healing. In this study, the ratio of wall thickness to density in the three-dimensional model of CT during bone healing can be used as a window to observe the healing process.

To simulate the process of bone healing, the influencing factors, and the degree of fracture healing, scholars have used the load analysis method for the above research. However, in the analytical process, the tissue needs to be simply divided; this causes the model data to be distorted, and the complex simulation results in tedious calculations and a long operation time. This model cannot be applied universally. The wall thickness ratio method also uses pre-processing technology, but it does not need to modify the model data, which is beneficial to the calculation process. This method only analyzes the mechanism of the bone tissue in the target segment and only removes the artifacts and selects the same range of bone CT values in the process of individualized modeling. Thus, modeling, drawing a triangular mesh, and conducting a morphological analysis can be carried out. Without the need for bone material assignment and optimization of the model, the distortion of the model and the complexity of the analysis steps are greatly reduced, and the analysis time can be greatly shortened, making this method fast and simple. At the same time, with the improvement in the simulation accuracy, the CT can be modeled separately at different time points to reflect the continuous changes in the bone morphology and bone mineral density and can show the bone healing progress [22–27].

Image evaluations of bone healing are mainly based on X-ray or CT images, which usually include bones, callus or trabecular bridging fractures, the disappearance of fracture lines, and continuity of the bone cortex. To quantitatively judge bone healing, in 2010, Whelan scored the callus and fracture lines on four sides of the tibia to judge the degree of tibial fracture healing via the tibial fracture imaging healing score (RadiographicUnionScoreinTibialfractures, RUST), which is recognized by clinicians. Later, the hip fracture imaging score (RadiographicUnionScoreforHip, RUSH) and the radius fracture imaging score (RadiographicUnionScoreforRadius, RUSS) were used for hip and radius fracture healing processes, respectively. However, the imaging healing scoring system needs to be used to judge whether the callus is formed and the fracture line disappears, which is still subjective and semi-quantitative, thus having some limitations. Yu Aihong and Humbert Ludovic et al. used the wall thickness ratio technique to estimate the hip cortex thickness and density through clinical CT to predict the risk of fracture. Calvani Lino et al. used a retrospective cohort study to explore the relationship between increased maxillary alveolar concavity and reduced labial cortical bone thickness assisted by cone beam computed tomography (CT). A study by Minonzio J-G and other preliminary studies have shown a correlation with non-traumatic fractures in postmenopausal women based on ultrasound measurement of cortical thickness and porosity assessment. Although methods for quantitative analysis have been applied in the clinic, there are no systematic experimental studies to verify the effectiveness and accuracy of quantitative analysis [10, 20, 28–33]. In this study, animal experiments are used to verify the results of the wall thickness ratio analysis. This method is a quantitative and objective research and analysis process.

The experimental results show that the wall thickness ratio analysis can better reflect the maximum bearing trend of the bone segment, and this method has better simulation results and reliability for the target bone segment. Therefore, the wall thickness ratio analysis technique can better simulate the stress conditions of the fracture end. On the other hand, this method omits the debugging process of assignment and the load environment, and it analyzes the callus change process during bone healing by calculating the ratio to the healthy side in a software environment. The degree of bone healing and bone tissue strength in the process of bone healing can be effectively determined.

## The study limitations and the follow-up plan

This study is a single-center prospective study. Due to the limitations of research funds and time, the sample size of this study is limited. The conclusion of the study reveals a preliminary trend, but large-sample, multi-center experimental research is needed in the future.

## Data Availability

Any data that support the findings of this study are included within the article.
